# Nutrition in the management of peripheral arterial disease: should we pay more attention to what our patients eat?

**DOI:** 10.1016/j.jvssci.2021.11.003

**Published:** 2021-12-06

**Authors:** Christopher O. Audu, Alan Dardik

**Affiliations:** a Section of Vascular Surgery, Department of Surgery, University of Michigan, Ann Arbor, Mich; b Department of Surgery, Yale University, New Haven, Conn

Peripheral arterial disease (PAD) manifests with a wide range of symptoms, from mild claudication to critical limb-threatening ischemia with tissue loss. Symptom severity is often a direct result of the increased atherosclerotic vascular burden and diminished limb arterial outflow. Current management paradigms ranges from conservative medical optimization that relies on antiplatelet and cholesterol lowering agents to invasive surgical intervention using endovascular, open, or hybrid approaches. However, this armamentarium is limited in its ability to encourage angiogenesis, especially when tissue loss has occurred. Contemporary attempts at inducing angiogenesis in patients with PAD using gene therapy have not yet been clinically efficacious.^1^^,^^2^ Thus, new therapeutic approaches to PAD are needed.

In a new *JVS Vascular Science* article, Tsuruoka et al^3^ found a unique role for an essential element, zinc. Zinc encouraged angiogenesis and limb salvage in a murine model of limb ischemia and improved surface perfusion in patients with PAD requiring surgical intervention. Normally, zinc is implicated in cellular oxidative processes and has not been linked to angiogenic properties in the PAD population. In their study, the authors showed that in diet-induced, zinc-deficient mice, the ischemic wounds exhibited slower, less-efficient repair and decreased angiogenesis compared with their wild-type counterparts consuming normal chow. They subsequently studied patients who had undergone an index surgical intervention for critical limb-threatening ischemia and noted that those with increased serum levels of zinc had better blood–tissue perfusion postoperatively, implying that zinc is essential to limb salvage.

The concept of nutrition in the context of surgical outcomes is not new.^4^^,^^5^ In general, well-nourished patients have better outcomes postoperatively because their intake matches the increased anabolic needs after surgery. The importance of trace elements such as zinc to complete nutrition has been shown by the requirement for its inclusion in total parenteral nutrition formulations.^6^^,^^7^ In addition, it has been recognized that patients with PAD taking certain antihypertensive medications such as an angiotensin receptor blocker, thiazide, angiotensin-converting enzyme inhibitor, or potassium-sparing diuretics might develop a net negative zinc balance that could necessitate monitoring and nutritional supplementation.^8^ What is unknown, however, is the role and mechanisms of trace elements, most of which serve as co-factors for essential enzymatic processes, in the management of PAD. Although much study has been performed on copper and selenium in cardiovascular disease,^9^^,^^10^ where they facilitate cell migration, proliferation, neurogenesis, and atherosclerosis, none of these has been studied in the context of PAD and ischemic wound healing. Thus, the study by Tsuruoka et al^3^ is particularly apt to our specialty.

In the end, could their findings change the pre- and postoperative treatment of patients with PAD? Potentially, yes; however, more research is needed to expand the current work. For one, detailed biochemical and immunologic mechanistic studies are required to fully understand zinc activity in angiogenesis. Additionally, better clinical outcome studies are needed, such as trials examining zinc supplementation for patients with PAD. Nevertheless, the work begun by Tsuruoka et al^3^ has provided a start towards a basic scientific underpinning for the validity of such research endeavors and potentially offers an alternative therapeutic strategy for a challenging disease.

*The opinions or views expressed in this commentary are those of the authors and do not necessarily reflect the opinions or recommendations of the Journal of Vascular Surgery* *Vascular Science* *or the Society for Vascular Surgery.*

## References

[1] Jazwa A., Florczyk U., Grochot-Przeczek A., Krist B., Loboda A., Jozkowicz A. (2016). Limb ischemia and vessel regeneration: is there a role for VEGF?. Vascul Pharmacol.

[2] Forster R., Liew A., Bhattacharya V., Shaw J., Stansby G. (2018). Gene therapy for peripheral arterial disease. Cochrane Database Syst Rev.

[3] Tsuruoka T, Kodama A, Yamaguchi S, Masutomi T, Koyama A, Murohara T, et al. Zinc deficiency impairs ischemia-induced angiogenesis. J Vasc Surg Vasc Sci . 10.1016/j.jvssci.2021.09.023 PMC8792263 35128488

[4] Whittle J., Wischmeyer P.E., Grocott M.P.W., Miller T.E. (2018). Surgical prehabilitation: nutrition and exercise. Anesthesiol Clin.

[5] Torgersen Z., Balters M. (2015). Perioperative nutrition. Surg Clin North Am.

[6] Michie D.D., MacFarlane M.D., Wirth F.H. (1977). Zinc and total parenteral nutrition. South Med J.

[7] Jeejeebhoy K. (2009). Zinc: an essential trace element for parenteral nutrition. Gastroenterology.

[8] Fenton R., Brook-Barclay L., Delaney C., Spark J., Miller M. (2016). Do medications commonly prescribed to patients with peripheral arterial disease have an effect on nutritional status? A review of the literature. Ann Vasc Surg.

[9] Kim B.E., Nevitt T., Thiele D. (2008). Mechanisms for copper acquisition, distribution and regulation. Nat Chem Biol.

[10] Liu H., Xu H., Huang K. (2017). Selenium in the prevention of atherosclerosis and its underlying mechanisms. Metallomics.

